# DRP lyase deficient DNA polymerase beta impairs mitochondrial electron transport chain and compromise mitochondrial DNA integrity

**DOI:** 10.21203/rs.3.rs-10361921/v1

**Published:** 2026-07-21

**Authors:** Dawit Kidane, Aashirwad Shahi, Nyima Kinteh, Shengyuan Zhao

**Affiliations:** College of Medicine, Howard University

## Abstract

Endogenous and environmental exposures can induce mitochondrial DNA (mtDNA) damage. Previous studies have shown that mtDNA is particularly vulnerable due to its proximity to mitochondrial reactive oxygen species (ROS) and the absence of histone-like protective proteins, both of which contribute to elevated levels of mtDNA damage. Base excision repair is a critical oxidative DNA damage repair pathway to reverse mitochondria genomic stability. In this work, we examined the impact and repair of ROS-induced DNA damage in the mtDNA due to lose of dRP layse activity of DNA polymerase beta (PolB). We used dRP lyase deficient DNA polymerase beta (PolB-dRP lyase) as a model to uncover the mechanism of mtDNA genomic instability and metabolic dysregulation. We have found that PolB-dRP lyase deficient cells significantly accumulate ROS, decrease mitochondrial encoding antioxidant genes, and low expression of genes involved in electron transport channels (ETC) including respiratory complexes I, II, III and IV. Further PolB-dRP lyase deficient cells exhibit a significant mtDNA damage and replication stress. Moreover, PolB-dRP lyase deficient stomach tissues of mice harbor a significant accumulation of ROS and alter mitochondria signaling pathways. Overall, this work highlights the molecular mechanism associated with PolB-dRP lyase function role in modulating ETC/ROS axis and maintaining mitochondrial DNA integrity.

## Introduction

Mitochondrial diseases are genetic disorders characterized by defects in metabolic pathways and arise from mutations in genes encoded by both nuclear DNA (nDNA) and mitochondrial DNA (mtDNA). It is estimated that the prevalence of mitochondrial diseases is one in five thousand cases and exhibits heterogeneous clinical features^1, 2^. Human cell contains hundreds to thousands of mitochondria depending on tissue and cell type, and each mitochondrion has multiple copies of mtDNA (1,000–10,000 copies/ cell)^3^, resulting in both homoplasmic and heteroplasmic variants. Moreover, the mtDNA copy number (mtDNAcn) is regulated in a tissue-specific manner^4^ and correlates positively with the number of mitochondria, and thus is considered an indicator of mitochondrial function^5^. Mitochondrial DNA is a circular double-stranded DNA and encodes 37 genes (13 proteins, 22 tRNAs, and 2 rRNAs)^3^. Most proteins required for mitochondrial structure, transport and energy production are encoded in the nucleus, but the mitochondrial genome is essential.

mtDNA is vulnerable to various environmental agents such as ultraviolet light, ionizing radiation (IR), chemicals, toxins and pollutants^6, 7 8^, as well as to endogenously generated alkylating agents and reactive oxygen species^9^. There are different types of mtDNA damage that originated endogenous or exogenous agent that result in alkylating mediated damage, oxidative damage, adducts formation, base mismatch and DNA strand breaks^10^. Given the proximity of mtDNA to ROS generating electron transport chain and the absence of histones, it is more vulnerable to oxidative DNA damage than nDNA^11^. Oxidative damage to mtDNA may be in the form of base modifications, abasic sites and various other types of lesions^12^. Excessive mtDNA damages, if not repaired efficiently, may increase ROS production, which in turn leads to mitochondrial dysfunction and provokes the pathogenesis of many human diseases^13, 14, 15^.

It is conceivable to speculate that the mtDNA could be a major target for ROS-mediated damage for several reasons. First, mitochondria do not have a complex chromatin organization consisting of histone proteins, which may serve as a protective barrier against ROS. Second, mtDNA has a limited repair activity against DNA damage. Third, a large part of O_2_^−·^, which is formed inside the mitochondria, cannot pass through the membranes and, hence, ROS damage may be contained largely within the mitochondria. Base excision repair (BER) is a major pathway to repair oxidative DNA damage in nuclear and mitochondria and its low or aberrant BER function leads to acceleration of genomic instability. BER defect leads to hypersensitive to endogenous oxidative stress that results in the formation of single strand breaks (SSBs) and double strand breaks (DSBs) when they encounter DNA replication^16^. Pol β plays a critical role in BER and non-homologous end joining repair pathways^17^. The Pol β is the first perform gap-filling DNA synthesis by its polymerase activity and then cleave a 5’-deoxyribose-5-phosphate (PolB-dRP) moiety via its PolB-dRP lyase activity^18^. DNA POLB and POLG are key DNA repair enzymes that are involved in mtDNA repair process^19^. The ability to effectively repair mtDNA damage is achieved through multiple, often overlapping, DNA repair pathways. The oxidative mtDNA damaged is recognized with different types of glycosylases and followed by base removal and further generate AP site, which is processed by APE1, the gap is filled by POLG with correct nucleotides. The Wilson study estimated that ~ 30% of POLB localizes to mitochondria through TOM20 colocalization studies^20^. Additional high-quality immunogold electron microscopy (EM) localization studies were performed on WT and *POLB* null MEFs, demonstrating ~ 20% mitochondrial matrix localization and 60% nuclear localization^21^. Several studies have shown that POLG can fill the mtDNA gaps and has weak PolB-dRP lyase activity^21^. In this case, POLB 5’ PolB-dRP lyase activity takes over to process the removal of the 5’-PolB-dRP group to undergo short patch (SP)-BER. The mtDNA nicks sealed with DNA LIG3, which is the only vertebral mitochondrial DNA ligase identified so far and essential for mitochondrial DNA maintenance and ensuring the completion of the DNA repair process^22, 23^.

Notably, Unrepaired DNA damage can give rise to nuclear and mitochondrial DNA associated genomic instability and induce signaling cascades leading to inflammatory responses^24, 25^. It is unknown that most of the repair proteins and/or enzymes are imported from nucleus and engage in processing of oxidative DNA in mtDNA damage via base excision repair (BER) pathway^26, 27, 28^. Loss of nuclear proteins significantly impaired the BER repair efficiency in mitochondria^29^. Therefore, regulation of mitochondrial BER is not expected to be independent of nuclear BER, though the extent to which DNA POLB mitochondrial BER is regulated with respect to mtDNA damage remains largely unknown. In the nucleus, the majority of 5’-deoxyribophosphodiesterase (5’-PolB-dRP lyase) activity comes from the core BER enzyme Polβ, and the activity has been reported to be a rate-limiting step in short-patch BER (SP-BER)^30^. Polβ is comprised of a catalytic domain required for nucleotide insertion and an 8-kDa N-terminal 5’-PolB-dRP lyase domain^30^. Considering that the 5’-PolB-dRP lyase activity of Polβ was measured to be 17-fold higher than the same activity in Polγ^31^, we sought to reinvestigate the potential impact of PolB-dRP lyase deficiency of the Polβ in the mitochondria genome integrity and ETC related outcomes. A deep understanding of the mechanisms underlying PolB-dRP lyase deficiency impact on mitochondria genome integrity and modulating the gene involved in ETC is critical. In this work, we found that PolB-dRP lyase deficiency result in accumulation of ROS and mtDNA oxidative damage, lower expression of antioxidant and ETC related genes in mouse stomach tissues. Our data suggested that PolB-dRP lyase function of PolB is required to keep the mitochondrial genomic integrity and prevent altered mitochondrial physiological functions.

## Results

### PolB-dRP lyase deficient cell accumulate ROS and suppressed antioxidants.

**Figure 1:**
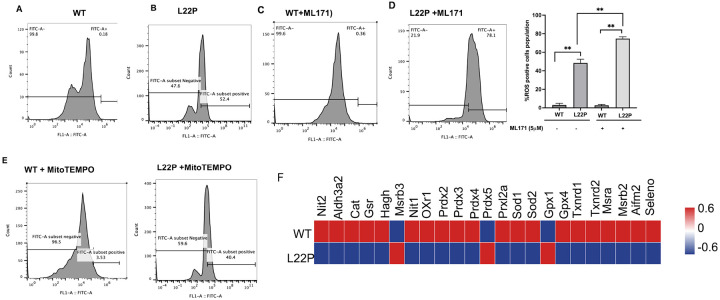
dRP lyase–deficient cells exhibit elevated ROS levels. (A–B) Representative histograms showing basal ROS in WT and dRP lyase–deficient cells.(C) ROS levels in WT and dRP lyase–deficient cells treated with NOX inhibitor ML171 (5 μM).(E) ROS levels following treatment ROS scavenger MitoTEMPO (10 μM) treatment in WT and dRP lyase–deficient cells.(D) The Bar graph shows the ROS level in untreated and ML171 treated WT and dRP Lyase-deficient cells. F) Heatmap of antioxidant defense genes expression from WT and L22P mice stomach tissue. Statistical comparison of the data was analyzed using t test with GraphPad Prism software (* P<0.05, ** P<0.01; *** P<0.0001).

Mitochondria are an important source of ROS production through the activity of the electron transport chain (ETC). Although there are also many other important sources of ROS generation (e.g. NADPH oxidases [NOX]), ETC-derived ROS are pivotal regulators of cell fate, given the central role of mitochondria in life and death. In this part of the study, we determined whether PolB-dRP lyase deficient cells accumulate ROS as compared to WT. Spontaneous accumulation of ROS in PolB-dRP lyase deficient cells are significantly high (52.4%) versus 0.18%; (P**<0.01Figure 1A and B). Further, we performed flow cytometry assay with and without antioxidant inhibitor including Nox inhibitor (ML171) and found that the number of cells with ROS significantly increase in PolB-dRP lyase deficient cells as compared to wild type cells (0.36% versus 78.1%; P**<0.01; Fig. 1C and D). Moreover, the level of ROS is reduced in PolB-dRP lyase deficient cells treated with MitoTempo to 40% (Figure E). Further, to determine whether DNA polymerase beta PolB-dRP lyase deficiency impact the gene expression of antioxidant, we performed RNA-Seq from RNA extracted from stomach tissues and found that PolB-dRP lyase deficient stomach harbor low level of antioxidant gene expression including PRDX2, PRDX3, PRDX4 PRDXl2a, SOD1, SOD2, TXRND1, TXRND2 CAT, (Fig. 1F).

### Loss of PolB-dRP lyase promotes downregulation gene expression involved in ETC

**Figure 2:**
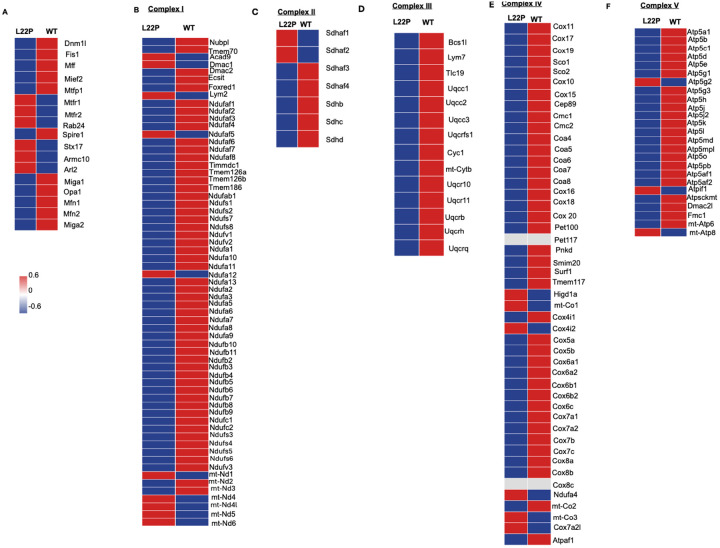
Loss of dRP lyase disrupts the expression of genes involved in mitochondrial dynamic genes and mitochondrial OXPHOS complexes I-V. (A) Heatmap of genes involved in mitochondrial fusion and fission process. (B-F) Representative Heatmaps of gene expression across complex I-V, showing coordinated suppression of OXPHOS subassembly modules genes across all five repository complexes.

In this study we investigated the impact of PolB-dRP lyase deficiency on mitochondrial dynamics by examining the expression of genes associated with fission and fusion. Transcriptomic analysis revealed that the genes involved in mitochondrial dynamics in PolB-dRP lyase deficient cells is inversely altered compared to WT (Fig. 2A). Fusion related genes Mfn1, Mfn2, Opa1, Miga1, and Miga2 are downregulated in PolB-dRP lyase deficient cells, while some fission related genes including Dnm1l, Fis1, Mff and Mief2 are similarly downregulated. In contrast, fission and mitophagy associated genes Mtfr1, Mtfr2, Armc10, Stx17, Rab24 is upregulated in PolB-dRP layse deficient cells. In the current study, we demonstrate that PolB-dRP lyase deficiency rapidly disrupts mitochondrial respiration by affecting gene expression of complexes I, II, III and V of the mitochondrial electron transport chain (ETC). Eight genes coding for the components of the mitochondrial ETC were identified along with 15 others related to mitochondrial function. Downregulation of NADH-ubiquinone oxidoreductase 20-kd subunit (ETC complex I), cytochrome c oxidase polypeptide Vic (ETC complex IV) and ATP synthase lipid-binding protein (ETC complex V) were further verified by real-time PCR. To determine the mechanism by which loss of PolB-dRP lyase cells harbor defect in OXPHOS complexes, we examined the mRNAs of individual mitochondrial and bioenergetic polypeptide genes. This revealed that loss of PolB-dRP lyase deficient cells exhibit suppressed the expression of groups of human genes associated with specific OXPHOS subassembly modules. Analysis of transcription in the L22P samples revealed that the gene expression of all five OXPHOS complexes (complexes I to V; Fig. 2B–F). Complex I incorporate seven mtDNA (MT-ND1, MT-ND2, MT-ND3, MT-ND4L, MT-ND4, MT-ND5, and MT-ND6) subunits and found that ND1, ND4, ND5 and ND6 expression upregulated in PolB-dRP lyase stomach tissues. In contrast ND2 and ND3 is down regulated (Fig. 2B). Further, Complex II is assembled from seven nDNA-coded subunits of succinate dehydrogenase encoding genes (SDHAF1, SDHAF2, SDHAF3, SDHAF4, SDHB, SDHC, SDHD) and five of them are downregulated (SDHAF3, SDHAF4, SDHB, SDHC, SDHD) and two (SDHAF1, SDHAF2) subunits are upregulated (Fig. 2C). Complex III encompasses one mtDNA subunit, MT-CYB, and 10 nDNA structural subunits and is assembled in stages are downregulated (Fig. 2D). For complex III, all nDNA structural genes were downregulated. Additionally, assembly factors UQCC1, UQCC2, and UQCC3, LYRM7, BCS1L, and TTC19 are downregulated (Fig. 2D). Complex IV incorporates three mtDNA subunits, MT-CO1, MT-CO2, and MT-CO3, each of which nucleates one of the three subassembly modules, using multiple assembly factors including *SCO2* (Fig. 2E). Complex V encompasses two mtDNA polypeptides, MT-ATP6 and MT-ATP8, and is assembled from five subassembly modules. For complex V, nearly all the nDNA genes in modules ATP5MC1, ATP5PB, and MT-ATP6, as well as the assembly factors DMAC2L and *TMEM70*, were downregulated, with the MT-*ATP8* gene being strongly upregulated. Last, four of the five structural genes of the ATPi5F1 and ATP5G2 module were up-regulated (Fig. 2F).

### Aberrant BER (L22P) causes the accumulation of mtDNA damage and decreases mtDNA copy number in vitro and in vivo

**Figure 3:**
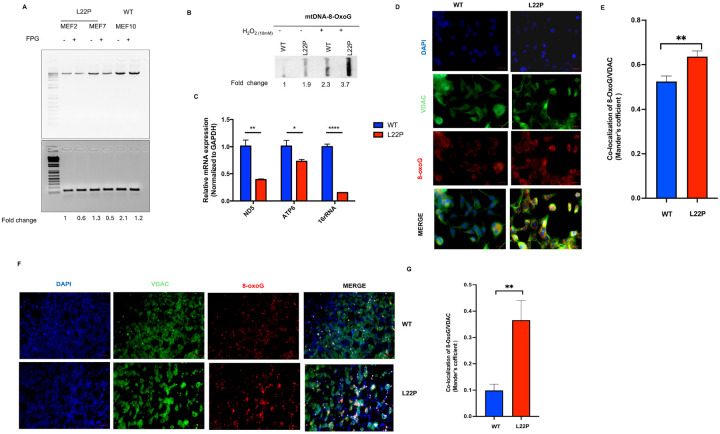
BER deficient leads to low mitochondrial copy number and increase oxidative mtDNA damage. A) mtDNA damage examined with long chain PCR after mtDNA treated with FPG glycosylase and the amplicon electrophoresis on agarose gel. B) Oxidative DNA damage was assessed by Slot blot analysis of 8-oxoG; C) mtDNA copy number measured using qPCR from WT and dRP lyase deficient stomach tissues of mice; D) Representative image of colocalization of 8oxoG with VDAC on MEFs cells derived from WT and dRP lyase deficient cells; E) Quantification of co-localization using as mander’s coefficient(tM2). F) Cellular localization of 8-OxoG and mitochondria from WT and dRP lyase deficient mice stomach tissues ; G) Quantitative analysis for the colocalization of the 8-Oxo-G with the VDAC expressed as mander’s coefficient(tM2). Note that tM2 represents the fraction of 8-OxoG signal overlapping with VDAC positive mitochondrial area. Statistical comparison of the data was analyzed using t test with GraphPad Prism software (* P<0.05, ** P<0.01;).

Our previously published data show that a POLB mutation in mouse embryonic fibroblast cells (MEFs) induces an accumulation of DSBs during the S phase of the cell cycle^33, 107^. Further, we have demonstrated that unrepaired DSBs progress into mitosis and generate chromosomal segregation defects^33^. However, it is still unclear as to the extent that endogenous oxidative mtDNA damage impacts the total mtDNA copy number in BER proficient versus deficient cells (POLB−/− and L22P). To address this issue, we analyzed mtDNA copy number using real-time quantitative polymerase chain reaction (RT-qPCR) from three different segments of the mitochondrial genome (ND5, ATP6 and 16rRNA) from DNA extracted from MEFs cells. We found that the mtDNA copy number significantly decreased in BER deficient cells versus wild type (WT) (Fig. 3A, P < 0.01, p < 0.5 and p < 0.001). To examine whether abrogation of BER exacerbates mtDNA genomic instability, we isolated mtDNA from BER deficient cells (POLB^−/−^, L22P) and BER proficient MEF cells and performed RT-qPCR to measure oxidative DNA damage analysis using a modified protocol, which measures the relative level of DNA strand breaks and/or polymerase-blocking lesions in a gene-specific manner. PCR based assay were performed after the genomic DNA was treated with formamidopyrimidine DNA glycosylase (FPG). FPG removes oxidative DNA lesions and processes abasic (AP) sites making single-strand nicks at oxidized bases or AP sites to produce two closely spaced single-strand nicks on opposing strands resulting in DSBs or blocks the amplification of any long PCR amplicon (13Kbp, Fig. 3B, left panel). A small DNA fragment of mtDNA (162bp) was also amplified to normalize the amplification of large fragment amplicons, as done before^108^. We found that oxidative mtDNA damage significantly increased approximately two-fold in, POLB^−/−^, L22P cells versus WT cells using RT-qPCR (Fig. 1B right panel). In addition, the DNA from BER deficient and proficient cells treated with FPG enzyme resulted in a 4-fold increase in mtDNA oxidative damage in BER deficient cells (ratio of WT FPG treated to L22P FPG treated). This result supports our finding of higher levels of oxidative lesions on mtDNA in BER deficient cells. Taken together, these data provide in vitro evidence that BER deficiency exacerbates the accumulation of DNA damage which is likely a driving force for inflammation under in vivo conditions. It is currently unclear as to the extent of interplay between BER deficiency and mitochondrial copy number. To address this issue, we measured mtDNA copy number in young (2 months old) and old (18 months old) mice using RT-qPCR analyses of DNA extracted from brain, liver and stomach as described previously^109^. We looked for three segments of mitochondrial genomic regions (ND5, ATP6 and 16rRNA). The number of mtDNA copies was significantly decreased in the brain, liver and stomach in BER deficient mice versus WT mice (Fig. 3C). Further, we also measured the level of DNA damage using RT-qPCR and found that oxidative DNA damage was significantly increased in tissues from BER deficient mice (Fig. 3C, P < 0.001). We also examined the mitochondrial oxidative DNA damage in WT and dRP lyase deficient mice by quantifying the colocalization of 8-oxoG with the mitochondrial outer membrane protein VDAC by immunofluorescence (Fig. 3D). Colocalization was quantified using the Mander’s coefficient tM2, representing the fraction of 8-oxoG signal overlapping with VDAC positive mitochondrial area. MEFs derived from dRP lyase deficient cells also showed increased mitochondrial 8-oxoG colocalization compared to WT (Fig. 3D and E, **P < 0.01). Consistent with this data, colocalization in stomach tissues derived from, dRP lyase deficient mice showed a higher tM2 compared to WT (Fig. 3F and G; P < 0.01). Together, these findings demonstrate that mitochondrial oxidative stress is elevated in dRP lyase deficient mice both in vivo and in vitro. Our results show that BER is essential to maintain mtDNA integrity in all three different tissues.

### PolB-dRP lyase deficiency triggers mtDNA replication.

**Figure 4:**
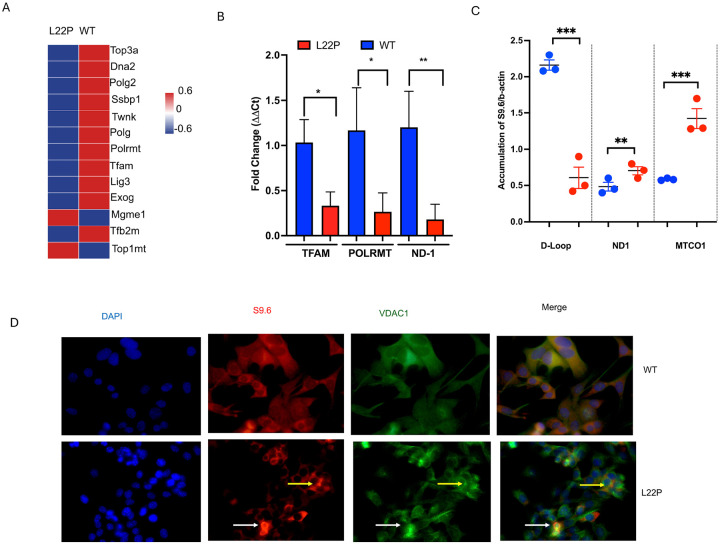
dRP Lyase deficiency impairs mtDNA replication (A) Heatmap showing downregulation of mtDNA replication and repair genes in dRP lyase deficient cell compared with WT cells. (B) RT-qPCR analysis shows downregulation of TFAM, POLRMT, and ND-1 in dRP lyase-deficient cells compared to WT controls.; C) DIP-qPCR data to measure DNA-RNA hybrid accumulation in the stomach tissues of WT and dRP lyase deficient stomach tissues of mice. D) Representative image of Immunofluorescence co-localization of S9.6 with mitochondria DVAC in WT and dRP lyase deficient cells Statistical comparison of the data was analyzed using t test with GraphPad Prism software (* P<0.05, ** P<0.01; *** P<0.0001).

To determine whether deficiency in PolB-dRP lyase function of DNA polymerase beta impairs mtDNA replication, we examined the transcriptional expression of several mt DNA replication and repair genes in WT and PolB-dRP lyase deficient stomach tissues (Fig. 4). Majority of mtDNA replication and repair genes including POLG, TWINKLE (DNA helicase), mitochondrial RNA Polymerase (POLRMT), mtSSB (mitochondrial single-stranded DNA-binding protein), RNASEH1, DNA ligase III, TOPO3A, TOPO1A, EXOG, and DNA2 are downregulated (Fig. 4A). Additionally, Top1mt and Mgme1 are upregulated in PolB-dRP lyase. Our RT-qPCR data also demonstrated genes like TFAM, POLRMT and ND-1 are downregulated in dRP lyase deficient cells (Fig. 4B, **P < 0.01). TFAM is a key regulator of mtDNA replication and packaging and POLRMT is sole RNA polymerase, where it generates primers for mtDNA replication.

Reduced expression of POLRMT and TFAM is expected to impair mtDNA replication and transcription. Consequently, decreased ND-1 expression (complex I-encoded gene) further indicates compromised mitochondrial gene expression. Furthermore, to examine whether DNA–RNA hybrids are involved in mitochondrial replication, we performed immunoprecipitation-based DIP-qPCR to quantify R-loop accumulation using the S9.6 antibody. The results revealed a significant shift in R-loop accumulation from the D-loop region to the ND1 and MTCO1 loci within the mitochondrial genome. These findings may indicate metabolic stress in dRP lyase-deficient stomach tissues (Fig. 4C; P < 0.0001, P < 0.001). In addition, we also performed immunofluorescence colocalization of S9.6 in mitochondria marker DVAC on WT and dRP lyase deficient cells (Fig. 4D). Our results demonstrate increased colocalization of mitochondrial RNA–DNA hybrids in dRP lyase-deficient cells, a phenomenon that may contribute to the induction of mitochondrial replication stress (Fig. 4D). Collectively, these findings indicate that loss of dRP lyase activity disrupts the coordination between mitochondrial transcription and translation, thereby compromising mitochondrial homeostasis.

## Materials and Methods

### Cell lines

We constructed a POLB L22P conditional knock-in mouse model as described previously^32^. C57BL/6 Mouse Embryonic Fibroblasts (MEFs) were isolated from embryonic tissue at embryonic day 14.5^32^. Two MEF cell lines isolated from WT and L22P mice were characterized. Embryos from WT and L22P transgenic mice were isolated at embryonic day 14.5. After the heads, tails, limbs, and most of the internal organs were removed, the embryos were minced and trypsinized for 20 min and then seeded into T-75 cell culture dishes in 10 mL DMEM supplemented with 10% fetal bovine serum (FBS), 1% penicillin/streptomycin, and 1% L-glutamine at 37°C with 5% CO_2_. The cells were split at 1:2 ratios when freshly confluent, passaged two or three times to obtain a morphologically homogenous culture, and then frozen or expanded for further studies.

### Measurement of ROS

The intracellular ROS was measured using 2’,7’-dichlorofluorescein diacetate (DCFDA) fluorescence. WT and DP16 MEFs cells were plated in a 96-well were stained with DCFDA (20 μM; Sigma) at 37°C for 2 h, and then fluorescence intensity was measured at the respective excitation and emission wavelengths of 485 nm and 535 nm using a fluorescent plate reader (HT-Synergy Agilent).

### Mitochondria DNA isolation and damaged qPCR

Cells were trypsinized and washed with PBS two times before DNA isolation. Mitochondrial DNA was isolated using QIAamp DNA Mini Kit (Cat. 51304, Qiagen) according to the manufacturer’s protocol. Oxidative DNA damaged was measured using long range PCR that using primers and internal control. mtDNA was treated with 20 units FPG (#M0240S, New England Biolabs) at 37°C for 3 h. mtDNA were resolved in 1% agarose gel, and the intensity of band were quantified using ChemiDoc Software (BioRad).

### DIP-qPCR

DRIP was adapted from the previously published procedure^33, 34^. WT and L22P mice stomach extract were resuspended in TE and lysed with 50 μl 20% SDS and 5 μl Proteinase K 20 mg/ml (ThermoFisher Scientific) for 3 h at 37°C. DNA was extracted by phenol–chloroform extraction using phase lock tubes and ethanol precipitated. Precipitated DNA was gently spooled and washed with 70% ethanol without centrifugation. DNA was allowed to air dry for 20 min and resuspended on ice in 130 μl TE buffer. DNA samples were subjected to a mechanical shearing using a Branson sonifier. The DNA samples were sonicated at 15% amplitude using a pulsed program consisting of 15 sec ON and 30 sec off for a total of 15 cycles to get a peak fragment size of 300 bp. Samples were maintained on ice throughout the sonication process. For RNase H-treated samples, 8 mg of DNA were treated with RNase H overnight at 37°C For each immunoprecipitation, 8 μg of sonicated DNA (with or without pre-treatment with RNase H treatment) was bound with 20 μg of S9.6 antibody (Abcam ,ab234957) in 1× binding buffer (10 mM NaPO_4_ pH 7, 140 mM NaCl, 0.05% Triton X-100) overnight at 4°C. Dynabeads Protein G beads (Thermo Fisher Scientific) were added for 2 h. Bound beads were washed three times in binding buffer and elution was performed in 250 μl elution buffer (50 mM Tris pH 8, 10 mM EDTA, 0.5% SDS, 8 μl Proteinase K 20 mg/ml) for 45 min with rotation at 55°C. The immunoprecipitated DNA was analyzed by qPCR. To calculate the percentage of input, apply the following formula for each locus: % input = 100*2^(Ct input(corrected) - Ct DRIPed DNA), where Ct (cycle threshold) input(corrected) = (Ct input - log2(10)) (we subtract log2(10) because the input represents 1/10th of the immunoprecipitated DNA).

### Flow cytometry assay

0.5 × 10^6^ per well of Wild type and dRP lyase deficient cells were seeded per well in a six well plate. Cells were treated with antioxidant inhibitors ML171– 5 mM and MitoTempo-10 mM for 24 hours. The total intracellular ROS level were then measured by staining the cells with 5 mM DCFDA dye for sixty minutes at 37°C. Following staining, cells were trypsinized, washed with FACS buffer for three times and, subjected to a flow cytometric analysis using a BD Accuri^™^ C6 plus flow cytometry. At least 10,000 events were recorded and analyzed using FlowJo software.

### Real-Time q-PCR

RNA was extracted using the Trizol/chloroform method and washed with 75% ethanol. cDNA was then immediately synthesized from RNA using High-Capacity cDNA Reverse Transcription Kit (Cat. 4368814, Applied Biosystems). To determine gene expression levels, synthesized cDNA was used as a template for real-time q-PCR using iTaq Universal SYBR Green Supermix (Cat. 1725121, Bio-Rad). Primers are listed in the PCR results were analyzed using 2^–ΔΔ^Ct method.

### RNA Extraction and Illumina RNA-Seq Library Preparation

Immediately after harvesting the samples, total cellular RNA was isolated from tissues using PureLink^™^ RNA Mini kit (Invitrogen, Cat#12183018A). The isolated RNA was cleaned with RNA clean & concentrator kit (Zymo research, cat#R1013). Then the RNA samples were quantified on a spectrophotometer (Multiskan SkyHigh; Thermo Scientific). The high-quality RNA samples were sent to Novogene corporation Inc. (Sacramento, CA, USA) for library preparation and Next-generation sequencing. The sequencing libraries were generated with TruSeq RNA Sample Prep Kit v2 Set A (Illumina). Briefly, poly(A) containing mRNA molecules were purified in two rounds using oligo(dT) attached magnetic beads from 1 *μ*g of total RNA. After chemical fragmentation, mRNA fragments were reverse-transcribed and converted into double-stranded cDNA molecules. Following end-repair and dA-tailing, paired-end sequencing adaptors were ligated to the ends of the cDNA fragments using TruSeq PE Cluster Kit v3-cBot-HS (Illumina).

### Sample preparation

Messenger RNA was purified from total RNA using poly-T oligo-attached magnetic beads. After fragmentation, the first strand cDNA was synthesized using random hexamer primers followed by the second strand cDNA synthesis. The library was ready after end repair, A-tailing, adapter ligation, size selection, amplification, and purification. The library was checked with Qubit and real-time PCR for quantification and bioanalyzer for size distribution detection.

### Clustering and sequencing

After library quality control, different libraries were pooled based on the effective concentration and targeted data amount, then subjected to Illumina sequencing. The basic principle of sequencing is “Sequencing by Synthesis”, where fluorescently labeled dNTPs, DNA polymerase, and adapter primers are added to the sequencing flow cell for amplification. As each sequencing cluster extends its complementary strand, the addition of each fluorescently labeled dNTP releases a corresponding fluorescence signal. The sequencer captures these fluorescence signals and converts them into sequencing peaks through computer software, thereby obtaining the sequence information of the target fragment.

### Bioinformatics Analysis Pipeline

We performed data quality procedure using raw data (raw reads) of fastq format were firstly processed through fastp software. In this step, clean data (clean reads) were obtained by removing reads containing adapter, reads containing ploy-N and low quality reads from raw data. At the same time, Q20, Q30 and GC content the clean data were calculated. All the downstream analyses were based on the clean data with high quality. In addition, reads mapping to reference genome were done. Briefly, reference genome and gene model annotation files were downloaded from genome website. Use HISAT2 (2.2.1) to build the index of the reference genome and use HISAT2 to align paired-end clean reads to the reference genome. HISAT2 can use the gene model annotation file to create splice-aware alignments, providing better alignment accuracy compared to other non-splice alignment tools.

### mRNA Expression by Real-Time-PCR (RT-PCR)

To evaluate the expression levels of selected genes by RT-PCR, 1 *μ*g of DNA-free total RNA isolated from the liver of the WT and DP16 mice was used for first strand cDNA synthesis with High-capacity cDNA Reverse transcription kit (Applied biosystems, cat#4368814)) using oligo(dT) and random primers. Quantitative polymerase chain reaction (qPCR) analysis was performed using PowerTrack^™^ SYBR Green Master Mix (Applied Biosystem, cat#A46109) with three technical replicates for each biological replicate, according to the manufacturer’s recommendation in a Quant studio 3 Real-Time PCR System (Applied Biosystem). Amplification was real-time-monitored and allowed to proceed in the exponential phase, until fluorescent signal reached a significant value (Ct). The fold change was determined using the 2^−ΔΔC(t)^ method^35^.

### In vivo mice study

All animal studies were conducted according to protocols approved by the Institutional Animal Care and Usage Committee of The Howard University (protocol # MED-02–23).

### Statistical analysis

Three independent experiments were performed for immunofluorescence, comet assay, AP site measurement and qRT-PCR. Data were statistically analyzed using Student t-test. Data from more than two study groups were analyzed using two-way ANOVA statistical analysis. Furthermore, the expression of PARP1 and interferon gene correlation was calculated using spearman coefficient with Graph Pad Prism software. Results were considered significant at P < 0.05.

## Discussion

Efficient and accurate replication and repair of mitochondrial DNA is essential for cellular viability due to its critical role in cellular energetics. Defects in mtDNA or the nuclear genes involved in mtDNA maintenance are associated with a variety of mitochondrial disorders^36^. mtDNA is susceptible to chemical and physical factors from endogenous and environmental sources and is maintained through DNA repair, turnover and the redundancy of mtDNA copies^37, 38^, in conjunction with mitochondrial dynamics (fission and fusion). Mitochondria are key contributors to total ROS levels and their impairment or cell stress can dramatically increase ROS production^39^. Our study shows that ROS levels increase significantly in PolB-dRP lyase deficient cells and inhibition of NOX as well as MitoTempo exacerbate the ROS level (Fig. 1). In addition, our data depict that the expression of antioxidant gene significantly compromised in PolB-dRP lyase deficient stomach tissues suggested that altered redox signaling that may impact mitochondrial dynamics. In particular, ROS may change mitochondrial fusion and fission related genes is significantly reduced in PolB-dRP lyase deficient stomach tissues, suggesting impaired mtDNA complementation through the mixing of damaged and intact mitochondrial genomes as well as defect in the segregation of mitochondria harboring damaged mtDNA from healthy mitochondria, potentially compromising the maintenance of mitochondrial integrity.

Mitochondrial fission and fusion are highly regulated and dynamic processes that remodels the mitochondrial network to meet the bioenergetic and biosynthetic demands of the cell. The dysregulation of fission and fusion dynamics leads to structural fragmentation or hyper fusion resulting in compromised Electron transport chain, oxidative stress and impaired cellular homeostasis. Our data shows that L22P mutation induces transcriptional reprograming of genes involved in fission and fusion relative to WT. Fusion related genes including MFN1, MFN2, OPA1, MIGA1, and MIGA2 are downregulated in PolB-dRP deficient cells, indicating compromised mitochondrial network connectivity and defects in cristae. Similarly, fission related genes DNM1L, FIS1, MFF, MIEF2, are upregulated while interestingly some genes involved in fission as well as autophagy including ARMC10, STX17 and RAB24 were upregulated. This shows that the PolB-dRP lyase deficiency not only impairs the fission fusion dynamics but also diverts the cell toward mitophagy clearance. Mitochondrial respiratory complexes are broadly disrupted in PolB-dRP lyase deficient stomach tissues. Within Complex I, mitochondria-encoded subunits (*mt-NAD1, mt-NAD4, mt-NAD5*, and *mt-NAD6*) are upregulated, whereas *mt-NAD2* and *mt-NAD3* are reduced, indicating altered subunit stoichiometry. Consistent with impaired assembly, expression of multiple Complex I assembly factors and accessory subunits (TMEM70, TMEM186, NDUFS1, NDUFV1, NDUFV2, NUBPL, NDUFAF5–7) is decreased, which is part of the respiratory chain and an important component of super complexes^40, 41^ and serves as one of the entry points for electrons into the OXPHOS system^42, 43^. Moreover, Complex II (CII) facilities bidirectional catalytic activity: including the peripheral catalytic subunit SDHA mediated interconversion of succinate and fumarate, while membrane subunits SDHC and SDHD facilitate electron transfer^44, 45, 46, 47^. Complex II–associated genes (SDHAF3, SDHB, SDHC, and SDHD) are similarly reduced, changes predicted to favor increased ROS production and inflammatory metabolic reprogramming. In contrast, SDHAF2, associated with CII subassemblies, is relatively increased, further supporting defective complex assembly. Results from this work shows that expression of both assembly and functional genes of CIII is significantly reduced in PolB-dRP lyase deficient stomach tissues, suggesting that CIII deficiency may likely contributes to elevated ROS levels. Our data is consistent with previously reported CIII function that has a high capacity for ROS production due in part to its ubiquitous and high expression^39^ and influence ROS mediated intracellular signaling^48, 49^. Consistent with broader mitochondrial dysfunction, expression of Complex IV subunits is also imbalanced, with increased mt-Co1 and mt-Co3 but reduced mt-Co2. Complex V displays analogous dysregulation, including altered expression of *mt-Atp6* and *mt-Atp8*, changes expected to compromise ATP synthesis and cellular energy homeostasis. This imbalance is likely to negatively impact mitochondrial bioenergetics universal energy currency highlighting the potential consequences of its dysregulation on cellular energy homeostasis^50^. Together, these data support a model in which PolB-dRP lyase deficiency drives coordinated disruption of respiratory complex assembly and stoichiometry across the electron transport chain, resulting in global mitochondrial dysfunction rather than isolated defects within individual complexes.

Notably, the change of metabolic alteration and ROS accumulation in PolB-dRP lyase deficient cell may contribute to mtDNA instability. DNA polymerase beta PolB-dRP lyase function is critical mitochondrial genome integrity including to protect the genome from oxidative stress associated mtDNA damage and replication stress. Results from this work pointed that DNA polymerase beta deficiency in PolB-dRP lyase function accumulate high level of oxidative DNA damage (Fig. 3). Several lines of evidence as well our current study suggested that lack of histone and the proximity of mtDNA to the main sites of mitochondrial ROS generation likely contribute to the higher steady-state levels of oxidative lesions and the higher instability observed in mtDNA when compared to nuclear DNA^51, 52, 53, 54^. In agreement with these observations, mtDNA copy number is significantly reduced in PolB-dRP lyase–deficient stomach tissues, whereas nuclear DNA content is largely preserved. RNA-seq analysis further reveals marked suppression of genes involved in mitochondrial DNA maintenance and replication, including TFAM and POLG. Core components of the mtDNA replisome mtDNA helicase (Twinkle),^55, 56^ mitochondrial single-strand DNA-binding protein (mtSSB),^57^ and the heterotrimeric replicative polymerase Polγ^58, 59^ also show significantly reduced expression in PolB-dRP lyase deficient tissues. In addition to Polγ, several auxiliary polymerases, (PrimPol, DNA polymerase β, DNA polymerase θ, and DNA polymerase ζ) have been reported to play a role in mitochondria replication^20, 60, 61, 62^. The PolB-dRP lyase activity of POLB likely substitute the removal of 5’-PolB-dRP group from the DNA nick to facilitate the polymerase activity of POLγ. Our in vivo data further suggest that reduced mtDNA copy number in PolB-dRP lyase deficient cells may result from ROS-induced mutations at mtDNA replication origins or lesions elsewhere in the genome that interfere with mtDNA replication^63^. It is also possible that accumulation of unrepaired mtDNA damage and replication stress contribute to mtDNA topological structure of change that negatively impact replication. Our results also shows that mtDNA replication shift from D-loop accumulation to increased DNA-RNA hybrid at the ND1 and MTCO1 regions signifies a major functional redirection within the mitochondria. This often indicates a cellular response to stress or metabolic dysfunction in dRP lyase deficient stomach tissues (Fig. 4C). Consistent with this model, recent studies highlight the importance of mitochondrial TOP1 (TOP1mt) in mitochondrial gene transcription^64^ and replication^65, 66^ and in maintaining mitochondrial function^67^. Our data suggest that the DNA polymerase plays a key role in DNA repair and is required to protect mitochondrial DNA integrity.

## Conclusions

In summary, our data suggest that PolB-dRP lyase deficiency induces mtDNA oxidative DNA damage and replication stress associated genomic instability. This study directly implicates DNA polymerase beta role in in mtDNA maintenance, likely via its removing 5PolB-dRP group from the DNA end. These results provide the mitochondria are proficient in BER pathway that may be important in promoting mtDNA stability and in preventing mitochondrial dysfunction and disease. Results from this work expand the understanding of the molecular mechanism by which metabolic dynamics including complex1, II, III, IV and V compromised in DNA polymerase beta deficient cells. This study advances our understanding of how mt DNA oxidative DNA damage and replicative stress in PolB-dRP lyase deficient cells drive dysregulated metabolic dynamics, offering new insights into the pathogenesis human disease.

## Supplementary Material

Supplementary Files

This is a list of supplementary files associated with this preprint. Click to download.

- SupplementFigure3CB.pdf
- LegendsSupplementFigure3.pdf

## Data Availability

All RNA-Seq data available and will be deposited in publicly available database. Other original data available for reanalyze the data reported in this paper available at the lead contact Dawit Kidane (dawit.kidane-mulat@howard.edu).
